# Machine-Learned Free Energy Surfaces for Capillary Condensation and Evaporation in Mesopores

**DOI:** 10.3390/e24010097

**Published:** 2022-01-07

**Authors:** Caroline Desgranges, Jerome Delhommelle

**Affiliations:** 1 MetaSimulation of Nonequilibrium Processes (MSNEP) Group, Tech Accelerator, University of North Dakota, Grand Forks, ND 58202, USA; cdesgr@gmail.com; 2 Department of Chemistry, University of North Dakota, Grand Forks, ND 58202, USA; 3 Department of Biomedical Engineering, University of North Dakota, Grand Forks, ND 58202, USA; 4 School of Electrical Engineering and Computer Science, University of North Dakota, Grand Forks, ND 58202, USA

**Keywords:** capillary, phase transition, free energy, activated process, liquid bridges, bubbles, machine learning

## Abstract

Using molecular simulations, we study the processes of capillary condensation and capillary evaporation in model mesopores. To determine the phase transition pathway, as well as the corresponding free energy profile, we carry out enhanced sampling molecular simulations using entropy as a reaction coordinate to map the onset of order during the condensation process and of disorder during the evaporation process. The structural analysis shows the role played by intermediate states, characterized by the onset of capillary liquid bridges and bubbles. We also analyze the dependence of the free energy barrier on the pore width. Furthermore, we propose a method to build a machine learning model for the prediction of the free energy surfaces underlying capillary phase transition processes in mesopores.

## 1. Introduction

The formation of liquid bridges and vapor bubbles between solid surfaces has drawn considerable interest. This is due to their significance in interface science and adhesion [1,2,3,4,5,6,7] and, in the case of nanoscopic capillaries, for their applications in nanotribology and nanolithography [8,9,10,11,12,13,14,15,16,17]. Density functional theory calculations [18,19,20,21] and molecular simulation studies [22,23,24,25,26,27,28,29,30,31,32,33,34,35,36,37,38,39,40,41,42,43,44,45] were instrumental in furthering our understanding of the formation of liquid-like junctions and of cavitation in nanopores. They also showed that the mechanism proposed for macroscopic capillaries proposed by Everett and Haynes [46] could be applicable to nanoscopic pores, and that, e.g., capillary condensation proceeds through a series of structural changes involving the formation of a liquid bridge across the pore section [37,39,45]. Here, we focus on combining machine learning (ML) with enhanced sampling simulations to provide a complete characterization of the capillary condensation and evaporation processes in cylindrical nanopores.

In recent years, ML emerged as an extremely useful tool to explore and predict complex phenomena [47,48,49,50]. Data-driven methods showed excellent results when applied to the identification of new force fields and coarse-grained models [51,52,53,54,55,56], the reconstruction of complex high-dimensional potential energy surfaces [57,58], and the prediction of thermodynamic and kinetic properties [59,60]. This considerably accelerates the determination of the key properties for these systems, since their computation via conventional molecular simulation methods often requires an extensive sampling of the phase space, i.e., performing simulations over very large time- and length-scales that quickly become extremely computationally intensive. Machine learning can also lead to new insights into assembly processes [61] and yield predictive models for heterogeneous catalysis [62]. Artificial neural networks were shown to provide a new way to obtain free energy landscapes that are difficult to compute. This is especially the case for processes that involve transitions from one state to another, a task for which rare event sampling and enhanced sampling simulations are required [63,64,65,66,67]. In the field of adsorption, ML techniques are also used to predict adsorption isotherms [68] and free energies of adsorption [69], catalytic activity [70], and to screen materials for gas storage and separation [68,71,72]. For instance, ML predictions on gas adsorption capabilities based on crystal designs such as MOFs and COFs at operating conditions [73,74,75,76] give excellent results when compared to that of conventional Monte Carlo and Molecular Dynamics simulations. They also provide unique routes to tailor new materials to enhance adsorption capabilities [77,78,79].

Here, we focus on the phenomena of capillary condensation and evaporation in nanopores. Such processes rely on the transition from a vapor phase to a liquid phase (condensation), or alternatively, the transition from a liquid phase to a vapor phase (evaporation). As a result, the system needs to overcome a free energy barrier to undergo a phase transition in these confined geometries [80]. Indeed, capillary condensation and evaporation originate from a metastable state and occur through an activated process, i.e., through a heterogeneous nucleation event [18,33,34,35,43,44,45]. To study these transitions and the formation of liquid bridges and bubbles during condensation and evaporation they entail, different molecular simulations techniques, such as Monte Carlo Gauge-cell methods, Expanded Wang–Landau algorithms or NPT-S approaches, were employed [35,45,81,82,83] on nanotubes of different natures, e.g., hydrophobic or hydrophilic [84]. These studies demonstrated that the capillary condensation process starts with the nucleation of a liquid bridge across the pore, which gradually extends through the length of the nanopore until the entire pore is filled with a liquid-like phase [39]. Similarly, the capillary evaporation process begins with the nucleation of a vapor bubble across the pore, which gradually extends through the length of the nanopore until the pore is completely filled with a vapor-like phase [39]. While recent progress shed light on the mechanisms underlying capillary phase transitions [35,45,81], the dependence of the process on the pore features is yet to be fully understood [85,86,87]. Most notably, the characteristics of the free energy barrier that controls the nucleation events, and thus, the capillary phase transition processes and its dependence upon pore width, are yet to be determined. We recently proposed a new simulation technique, termed as the μVT-S simulation method, based on an entropic reaction coordinate to elucidate the nucleation pathway [45,88,89] and apply it here to the case of capillary phase transitions in pores of increasing diameters. After identifying the free energy profile for the capillary phase transition processes through simulations for a few sets of conditions, we develop an ML model that generalizes the prediction of the free energy profile to a broad range of conditions.

In this paper, we focus on generating data using the μVT-S simulation technique to obtain free energy surfaces for the condensation and evaporation in nanopores of various geometries. Then, we use machine learning and, more particularly Artificial Neural Networks (ANNs), to predict free energies for a wide range of nanopore width and length. The paper is organized as follows. In the next section, we present the force fields used to model the adsorbed gas and the cylindrical nanocapillaries. We also give a brief account of the simulation method and technical details employed in this work. We provide the main conclusions from this work in the last section.

## 2. Simulation Method and Models

### 2.1. Force Fields

We use a Lennard–Jones potential to model the interactions between Argon atoms through

(1) $$\phi \left(r_{ij}\right)=4\epsilon_{ij}\left[{\left(\frac{\sigma_{ij}}{r_{ij}}\right)}^{12}-{\left(\frac{\sigma_{ij}}{r_{ij}}\right)}^{6}\right]$$

with the following parameters: m=39.95 g/mol, σ=3.4 Å and ϵ=119.8 K. This force field was shown to model very well the fluid properties for the liquid and vapor phases, as well as the vapor-liquid equilibria. It was also used to study adsorption in different materials, including nanotubes, MOFs, and COFs, leading to a very good agreement between simulation results and experimental data [39]. We use the conventional system of reduced units [90], in which σ is the unit length, ϵ is the unit energy and *m* the unit mass.

The pores we model are MCM-41 silica mesoporous molecular sieves. As shown in prior work by Neimark et al. [39], this model provides an excellent agreement for Argon adsorption isotherms between simulations and experiments. In this work, we consider cylindrical nanopores with 4 different pore widths (R=10σ, R=12σ, 16σ and 20σ) and a length of H=30σ (the axis of the cylindrical pore is along the *z* direction). The interactions between Argon atoms and the nanopore are modeled with a functional form $U_{sf}\left(r_{i},R\right)$, commonly used to model MCM-41 silica mesoporous molecular sieves [39,45,91], and given by

(2) $$\begin{array}{cc} U_{sf}\left(r_{i},R\right)= & \pi^{2}\rho_{s}\epsilon_{sf,i}\sigma_{sf,i}^{2}\times \\ \; & \left\{\frac{63}{32}{\left[\frac{R-r_{i}}{\sigma_{sf,i}}\left(1+\frac{r_{i}}{R}\right)\right]}^{-10}F\left[\frac{-9}{2},\frac{-9}{2};1;{\left(\frac{r_{i}}{R}\right)}^{2}\right]-3{\left[\frac{R-r_{i}}{\sigma_{sf,i}}\left(1+\frac{r_{i}}{R}\right)\right]}^{-4}\right.\\ & \left.F\left[\frac{-3}{2},\frac{-3}{2};1;{\left(\frac{r_{i}}{R}\right)}^{2}\right]\right\} \end{array}$$

in which $r_{i}$ is the radial coordinate of an argon atom *i* in the pore, *R* is the pore radius, $\rho_{s}$ is the surface density of adsorption centers and F(α,β;γ;δ) is the hypergeometric series. The parameters for the fluid-solid interactions are $\sigma_{sf,i}=3.17$ Å and $\rho_{s}\epsilon_{sf,i}$ 2253 K/nm${}^{2}$ [39,45]. We add that both the fluid model and the pore-fluid model were parametrized to provide an accurate account of the experimental data. The force field for the fluid was parametrized to model the experimental data for the phase transitions and boiling point for Argon, and the pore-fluid interaction was parametrized to model the experimental data for the adsorption of Argon in MCM-41 [91].

### 2.2. μVT-S Simulations

The formation of a liquid bridge during capillary condensation (or of a vapor bubble during the evaporation) are rare events since they are associated with configurations with a very low probability, and thus very rarely visited, when using conventional sampling methods. To capture the mechanism underlying the transition phenomenon, i.e., the transition from a metastable vapor to a stable liquid phase (condensation) or from a metastable liquid to a vapor phase (evaporation), one needs to enable the system to overcome the large free energy barrier related to the formation of either a bridge or a bubble. Several methods were developed to simulate nucleation processes. One of them, known as the umbrella sampling, relies on the use of a bias potential to overcome the free energy barrier and then to simulate the entire nucleation process [45,66,92,93,94,95,96,97,98,99,100,101]. The bias potential is a harmonic function of a reaction coordinate, which drives the nucleation process. For vapor → liquid and liquid → vapor transitions, it was suggested that entropy could be an efficient reaction coordinate [102], since it gives a thermodynamic measure of order and disorder and clearly distinguishes between the two phases. It is thus possible for the vapor → liquid nucleation process to induce order within the system, by decreasing the target value of the entropy in the umbrella sampling potential. By the same token, it is also possible to study the liquid → vapor transition by increasing entropy. Here, we carry out grand-canonical Monte Carlo (MC) simulations combined with the umbrella sampling technique using entropy as a reaction coordinate. As discussed in previous work [34,45,84], the umbrella sampling potential is of the form $V_{US}=\frac{1}{2}k{\left(S-S_{0}\right)}^{2}$, in which *k* is a spring constant, $S=\frac{U+k_{B}T\ln Q\left(N,V,T\right)}{T}$ the value taken by the entropic reaction coordinate for the configuration of the system, and $S_{0}$ the target value for the entropy imposed to the system. Here, since the system is confined in a nanopore, the product PV is very small compared to μN and thus can be neglected in the determination of *S* [45,102]. This leads to the following expression for the entropic reaction coordinate $S=\frac{U-\mu N}{T}$. We add that we use the total entropy $S^{*}$ as the reaction coordinate rather than, for instance, the number of atoms in the pore $N_{p}$ because $N_{p}$ does not characterize the onset of organization in the system, i.e., the nucleation of the liquid bridge in the case of capillary condensation. For instance, for the same $N_{p}$, the confined fluid can take the form of either a uniform fluid of intermediate density, or a fluid with a region of high density (liquid bridge) and a region of low density (surrounding vapor-like fluid), or a fluid with multiple small liquid-like clusters surrounded by a vapor-like fluid as a result of “entropic breaks” [103]. For this reason, and in line with recent work in the field of nucleation by, among others, Parrinello and co-worker [104,105] and by our group [45,66,88,89], we employ an entropic reaction coordinate. As shown later in Figures 3 and 4, using $S^{*}$ allows for a steady growth in the width of the liquid bridge that nucleates and extends across the pore and, as a result, in the number of atoms in the pore. We add that, in prior simulation work, Neimark and coworkers [38,39,106] investigated the capillary phase transitions in nanopores, and the formation of liquid bridges during capillary condensation, using grand-canonical, gauge-cell and NVT Monte Carlo simulations. We tested in previous work [45] the μVT-S simulation method against the results obtained by Vishnyakov and Neimark [39]. This allowed us to show that the μVT-S simulation method provides the same mechanism, involving the nucleation of a liquid capillary bridge as an intermediate state along the capillary condensation pathway, as that identified Vishnyakov and Neimark under the same thermodynamic conditions (chemical potential and temperature). Technical details on the implementation of this method can be found in previous papers [45,84,88,89]. Each μVT-S simulation provides an histogram for the probability distribution of how often a given value for the entropy is visited during the simulation. From a practical standpoint, we first carry out an equilibration run of $50\times 10^{6}$ MC steps, followed by a production run of $100\times 10^{6}$ MC steps during which the entropy histogram is collected. Using 40 overlapping windows, and following Torrie and Valleau’s work [92], we can reconstruct the free energy barrier associated with the nucleation process. Let us add that the MC moves used in the simulations are as follows: (i) translation of a randomly chosen argon atom (50% of the attempted MC moves), (ii) insertion of an atom (25% of the attempted moves) and (iii) deletion of a randomly chosen atom (25% of the attempted moves). Simulations are performed for a temperature of 0.73 (87.454 K), which corresponds to the boiling point for Argon. Under these conditions, and as shown by Neimark et al. [38,91], capillary condensation and evaporation of Argon was observed in MCM-41. We add that, for bulk Argon, the vapor-liquid phase transition occurs for μ=−10.53 in reduced units. In line with prior work, we use a cutoff of 15 Å and periodic boundary conditions along the lateral direction *z*.

### 2.3. Machine-Learned Free Energy Surfaces

**Figure 1:**
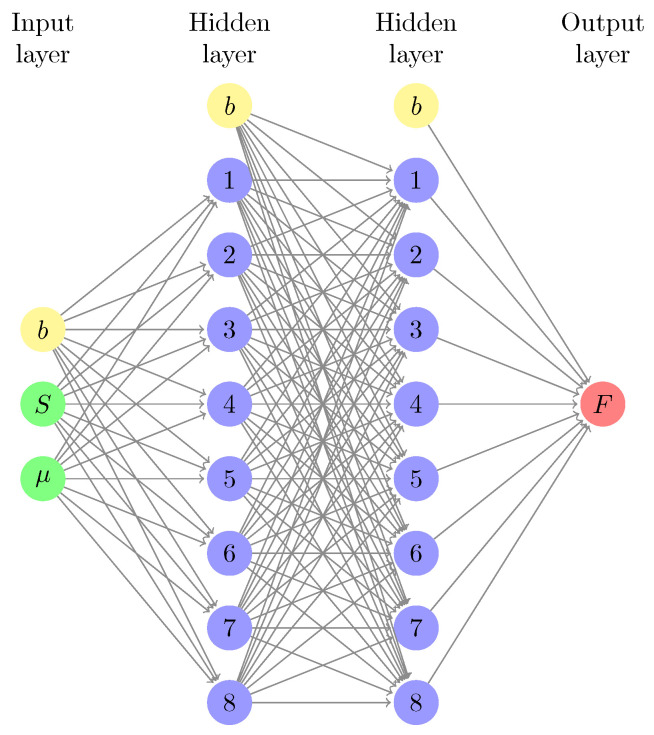
Artificial Neural Network (ANN) for prediction of free energy surfaces for capillary condensation and capillary evaporation.

Simulations require generating billions of configurations to sample the entire phase transition pathway for a single value of the chemical potential. To identify accurately the conditions of coexistence, simulations need to be repeated systematically for many chemical potentials, with a small chemical potential interval between successive runs. Given the cost of each simulation, we choose here to run simulations that have only a few values of the chemical potential and use the simulation results to generate a training dataset for an ML model. Once trained, the ML model has the advantage of being able to provide very rapidly the free energy profile for any value of the chemical potential. In other words, the ML model has the ability to interpolate between simulated conditions and to extrapolate beyond these conditions. Here, to generate machine-learned free energy surfaces for a given pore width, we use an artificial neural network (ANN) with a feed-forward structure. The ANN weights are optimized with a back-propagation algorithm [107]. We build on our previous work on the bulk thermodynamic properties of single-component systems and binary mixtures [108,109] to design an ML model that predicts the free energy surface of adsorption for a given pore width as follows. We optimize an ANN with 4 layers: (i) 1 input layers with 2 neurons for μ (chemical potential) and *S* (entropy), (ii) 2 hidden layers with $h_{1}=8$ and $h_{2}=8$ neurons, and (iii) 1 output layer with 1 neuron for *F* (Free energy). We then have the following analytic expression for the ML prediction for $F^{ML}$

(3) $$F^{ML}=f_{4}[b_{3}+\sum_{l=1}^{h_{2}}W\left(3,4,l,1\right)f_{3}\left(b_{2}+\sum_{j=1}^{h_{1}}W\left(2,3,j,l\right)f_{2}\left[\left(b_{1}+\sum_{i=1}^{3}W\left(1,2,i,j\right)G_{i})\right]\right)\right]$$

with **W** the weight matrix, $f_{1}$,$f_{2}$, $f_{3}$ and $f_{4}$ representing activation functions (tanh for the first three, and the linear function for the forth one), $b_{i}$ the bias nodes and $G_{i}$ the input neurons. The network architecture is summarized in Figure 1. The weight matrix **W** is initially filled with random numbers and optimized by minimizing the squared error function using a back-propagation algorithm and a learning rate of 0.04. μVT-S simulation results are split between training and testing data sets to optimize the ANN weights, with a training dataset size of about 10,000 data points for each pore width.

## 3. Results and Discussion

**Figure 2:**
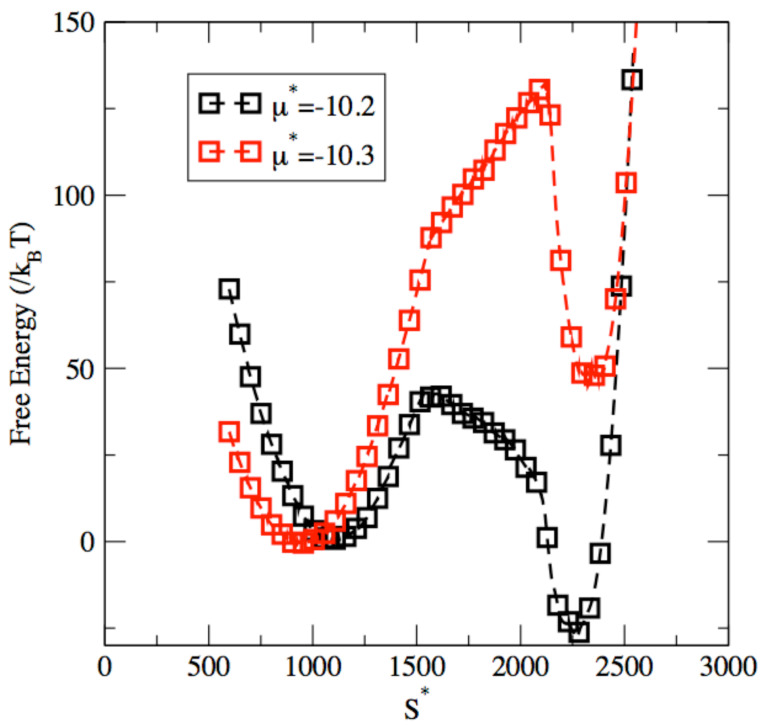
Capillary phase transitions in a nanopore with a pore width of 12σ: free energy against entropy for $\mu^{*}=-10.2$ (capillary condensation in black) and for $\mu^{*}=-10.3$ (capillary evaporation in red).

**Figure 3:**
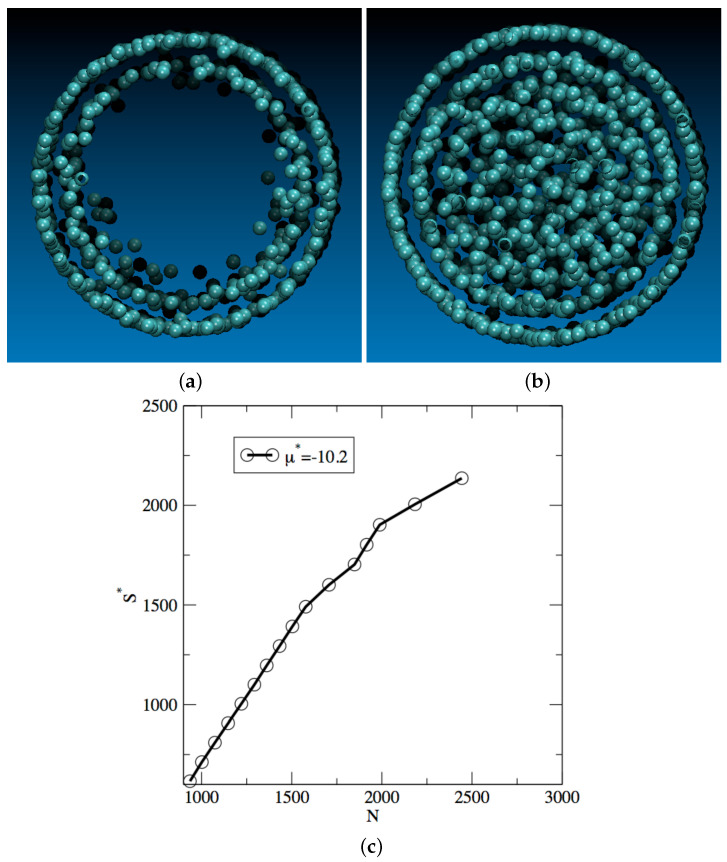
Snapshot of representative configurations for $\mu^{*}=-10.2$ for $S^{*}=1100$ (**a**) and $S^{*}=2250$ (**b**). (**c**) Shows variation of entropy as a function of number of atoms in pore.

**Figure 4:**
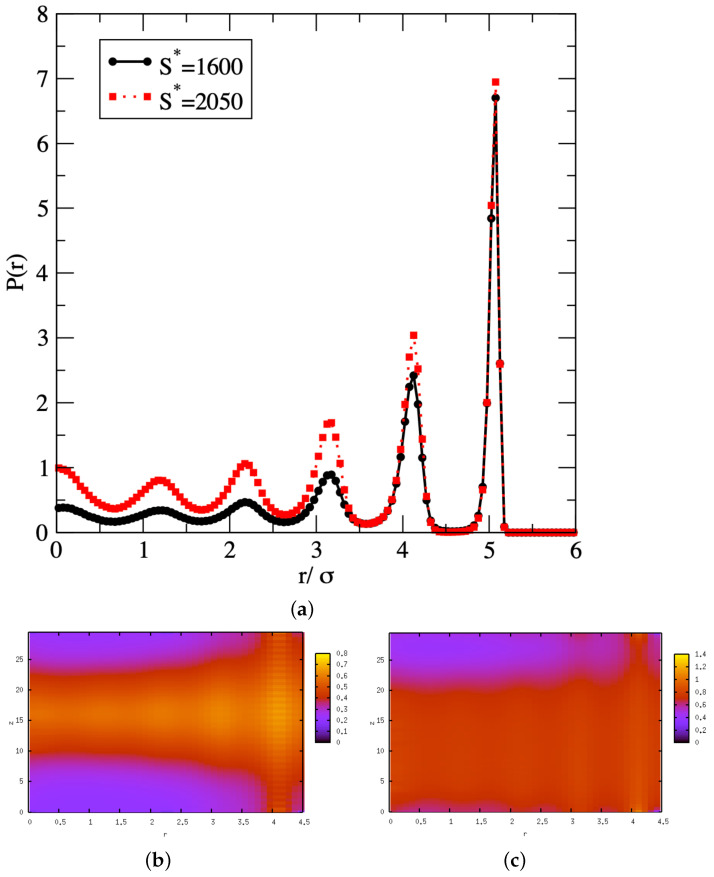
Radial density profile P(r) in a pore with a diameter of 12σ for $\mu^{*}=-10.2$ (**a**) and spatially resolved density distribution function n(r,Z) for $S^{*}=1600$ (**b**) and $S^{*}=2050$ (**c**).

We start by analyzing the results obtained from the MC μVT-S simulations on the example of a pore width of 12σ. We show in Figure 2 the free energy profiles reconstructed from the simulation results for two different values of chemical potential, $\mu^{*}=-10.2$ and $\mu^{*}=-10.3$. The free energy profile is plotted as a function of the reduced entropy of the adsorbed fluid $S^{*}$, which stands for the total entropy. In other words, this is an extensive quantity, a function of the number of Argon atoms adsorbed in the pore, and low values for the total entropy $S^{*}$ are associated with a low-density, vapor-like phase, while high values for the total entropy $S^{*}$ correspond to a high-density, liquid-like adsorbed phase. The plots exhibit two free energy minima, as well as a free energy barrier that connects the two minima. For each plot, the shallower minimum corresponds to the metastable phase that is the starting point for the phase transition process, and the deeper free energy minimum corresponds to the equilibrium state, i.e., the endpoint for the phase transition process. In between, and as expected for any nucleation process, the system has to overcome a free energy barrier to complete the phase transition process [93,96,100]. This feature, common to all nucleation processes, corresponds to an interplay between two contributions to the free energy of opposite signs. The first contribution has a positive sign and stems from the onset of a nucleus of the new phase, and thus from the creation of an interface that results in a free energy cost. The second contribution has a negative sign and results from the stabilization of the system, as the metastable phase of higher free energy undergoes the transition to the stable phase of lower free energy. As shown in previous work [34,39,45] and in Figure 3 and Figure 4, the free energy barrier is associated with the formation of a liquid bridge across the nanopore during capillary condensation and a vapor bubble across the nanopore during capillary evaporation.

The correspondence between the total entropy $S^{*}$ and the nature of the two phases can best be understood by examining snapshots of the system for the two minima exhibited by the free energy profile. Figure 3a shows a snapshot for a configuration of the system observed for $S^{*}=1100$, i.e., for the shallower minima in the free energy profile. The configuration shows that the pore is coated with two layers of the adsorbed Argon fluid, with a very low fluid density on the inside of the pore. Since the low-density, vapor-like phase is associated with the shallower free energy minimum for $\mu^{*}=-10.2$, this means that this phase is metastable and that the low-density phase, akin to a supersatured vapor, is the metastable phase for this set of conditions. On the other hand, Figure 3b shows a snapshot for a configuration of the system observed for the deeper free energy minimum, reached for a total entropy of $S^{*}=2250$. This snapshot shows that a completely filled up pore, corresponding to a high density liquid-like phase for the adsorbed fluid. Since this high density phase is associated with the deeper free energy minimum, this means that the liquid-like phase is the stable phase under those conditions. For a chemical potential $\mu^{*}=10.2$, we thus have a low-density, vapor-like, metastable phase and a high-density, liquid-like, stable phase, which implies that the free energy profile shown in Figure 2 for $\mu^{*}=-10.2$ corresponds to a capillary condensation process. Conversely, for $\mu^{*}=-10.3$, the deeper free energy minimum is obtained for a total entropy of about 1000, corresponding to the stable low-density, vapor-like phase, and the shallower free energy minimum is reached for a total entropy of about 2300, which is associated with a metastable high-density, liquid-like, adsorbed phase. This implies that, for $\mu^{*}=-10.3$, the system undergoes a capillary evaporation process as the total entropy decreases.

To identify the microscopic mechanisms underlying the phase transition process, we carry out a series of structural analyses for the adsorbed fluid. For this purpose, we focus on the capillary condensation process and analyze the structure of the adsorbed fluid for a chemical potential of $\mu^{*}=-10.2$ and a pore width of 12σ. We show in Figure 4a the radial density profile obtained for conditions close to the two “breaks” observed in the free energy profile. For the first “break”, i.e., for $S^{*}=1600$ and close to the free energy maximum during the phase transition process, we observe the formation of a third peak in the radial density profile at a distance of about 3.1 Å from the central axis of the pore, and the onset of a couple of more peaks as the distance from the central axis decreases. This first “break” therefore indicates some structural change is induced by a partial filling of the pore. For the second “break”, i.e., for $S^{*}=2050$ and close to the free energy valley attached to the stable high-density, liquid-like phase, we can see 5 peaks in the radial density profile, in addition to a developing peak in the center of the pore. The second “break” thus corresponds to an almost complete filling of the pore. We also provide in Figure 3c a plot of the entropy as a function of the number of atoms in the pore. We recall that $S^{*}$ is an extensive property and, as such, the increase we observe in Figure 3c for $S^{*}$ is in line with the expectations. We add that $S^{*}$ does not vary strictly linearly with *N*, since $S^{*}$ provides a quantitative measure of the amount of organization within the confined fluid as its density increases.

To provide further insight into the filling mechanism, we carry out a higher-resolution analysis and compute a spatially resolved density distribution function n(r,z) along the radius of the pore *r* as well as along the lateral dimension of the pore *z*. This enables the spatial resolution of the developing peaks in the radial density profile of Figure 4a. This yields the plots shown in Figure 4b,c for $S^{*}=1600$ and $S^{*}=2050$, respectively. At $S^{*}=1600$, the plot in Figure 4b shows that the density distribution is far from uniform along the lateral dimension *z*. Instead, the plot shows that, for 10σ<z<25σ, the density of the adsorbed fluid remains fairly high for all *r* values, which means that for these *z* values, a liquid region reaches across the width of the pore. For other *z* values, i.e., z<10σ or z>25σ, the fluid density remains low for all *r* values below 3.5σ. This means that the liquid region is surrounded by a vapor-like fluid. Therefore, the spatially resolved density distribution provide supporting evidence for the formation of a liquid bridge that extends across the capillary. At $S^{*}=2050$, the Figure 4c also shows a nonuniform density distribution along the lateral dimension *z*. Indeed, the liquid region now extends between 0σ<z<25σ, with a fluid density remaining high for all *r* values. On the other hand, the low density is restricted to a much smaller range of *z* values (z>25σ). This means that the liquid bridge now occupies the major part of the pore. These profiles thus provide an explanation for the two “breaks” in the free energy profile, with the first “break” corresponding to the onset of a capillary liquid bridge and the second “break” to the capillary liquid bridge taking over the entire pore. The simulation results show that the mechanism for capillary condensation starts with the nucleation of a liquid bridge, which subsequently grows and takes over the entire nanopore. This is in line with the mechanism proposed by Everett and Haynes for capillary condensation in micropores [46], and withe the machanism proposed by Neimark et al. for capillary condensation in nanopores [39].

**Figure 5:**
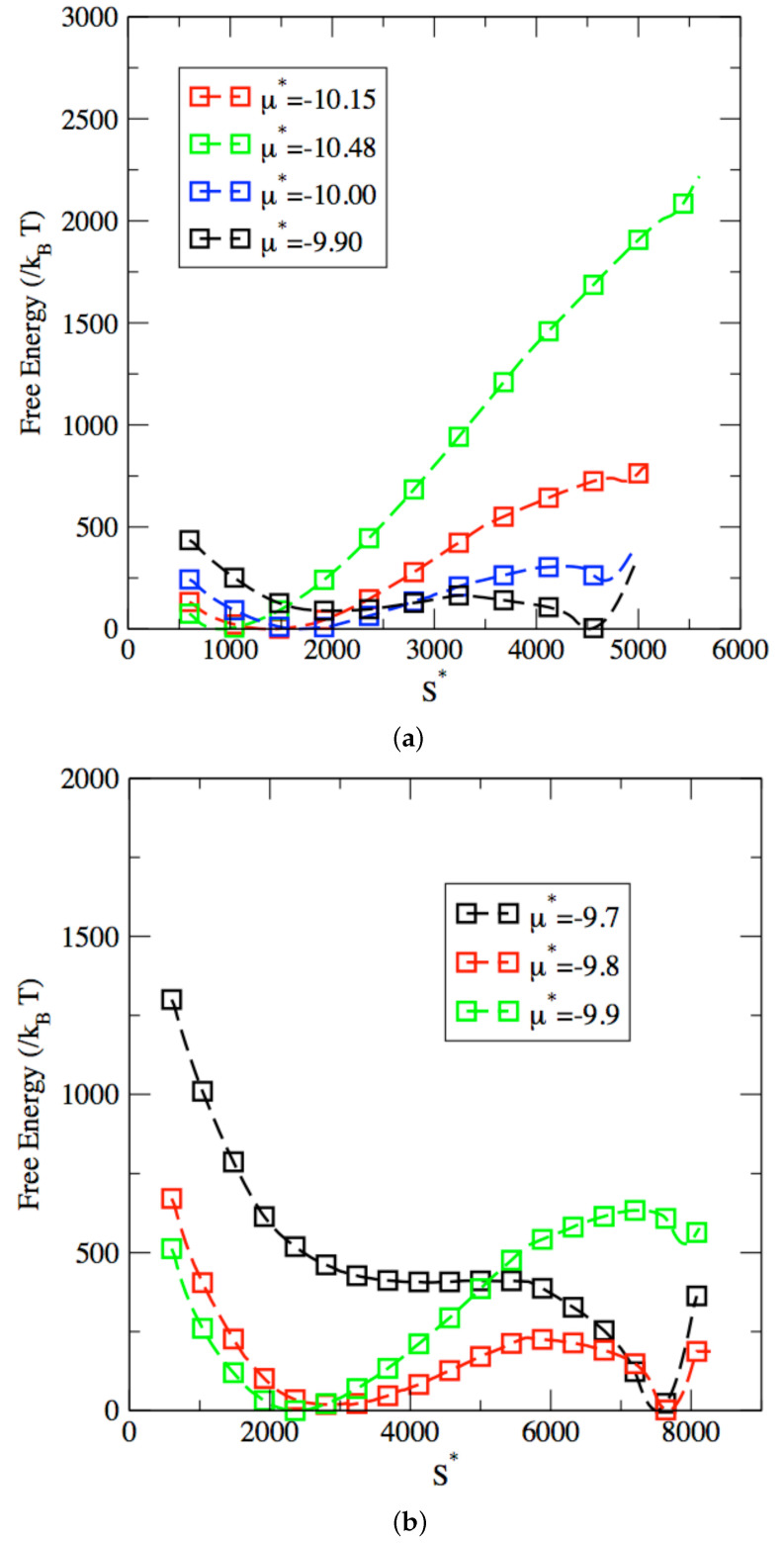
Comparison between free energy profile obtained for a pore width of 16σ (**a**) and 20σ (**b**). Results are shown for chemical potentials of $\mu^{*}=-9.9$ (black), $\mu^{*}=-10$ (blue), $\mu^{*}=-10.15$ (red) and $\mu^{*}=-10.48$ (green) in (**a**). Results are shown for $\mu^{*}=-9.7$ (black), $\mu^{*}=-9.8$ (red) and $\mu^{*}=-9.9$ (green) in (**b**).

We present in Figure 5 the free energy profiles obtained for the two largest pore widths of 16σ and 20σ. While similar filling mechanisms are observed for the different pore widths studied in this work, there are, however, three features of the free energy profiles that are impacted by the change in pore width. First, the path that joins the metastable and stable phases is much longer as the pore width increases. This translates into the much larger range of values for $S^{*}$ spanned as the width increases from 16σ, with $S^{*}$ ranging from 1500 to 4500 (see Figure 5a) to 20σ , with $S^{*}$ varying from 2500 to 7500 (see Figure 5b). This can be attributed to the fact that, for the same lateral length of the pore, many more fluid particles can be accommodated by the fluid, which in turn results in much larger values for the extensive property $S^{*}$. Second, the free energy barrier increases with the pore width. Since the filling mechanism involves the formation of a liquid bridge across the pore, as discussed earlier for the pore width of 12σ, this step will require overcoming a greater free energy for larger pores. Third, the chemical potential at coexistence is also impacted by the pore width. Coexistence is achieved for a chemical potential between −9.9 and −10 for a pore width of 16σ and of about −9.8 for a pore width of 20σ. This can be accounted for by the change in balance between the relative contribution of wall-fluid interactions and fluid-fluid interactions in the overall energy of the adsorbed phases. Indeed, coexistence here corresponds to two phases, one of low density and the other of high density, having the same chemical potential. As shown in Figure 3a for a pore width of 12σ, the low-density phase consists of two fluid layers adsorbed on the inner surface of the pore, which means that its chemical potential strongly depends on wall-fluid interactions. On the other hand, as shown in Figure 3b, the high-density phase is a completely filled pore, for which fluid-fluid interactions far outweigh wall-fluid interactions.

**Figure 6:**
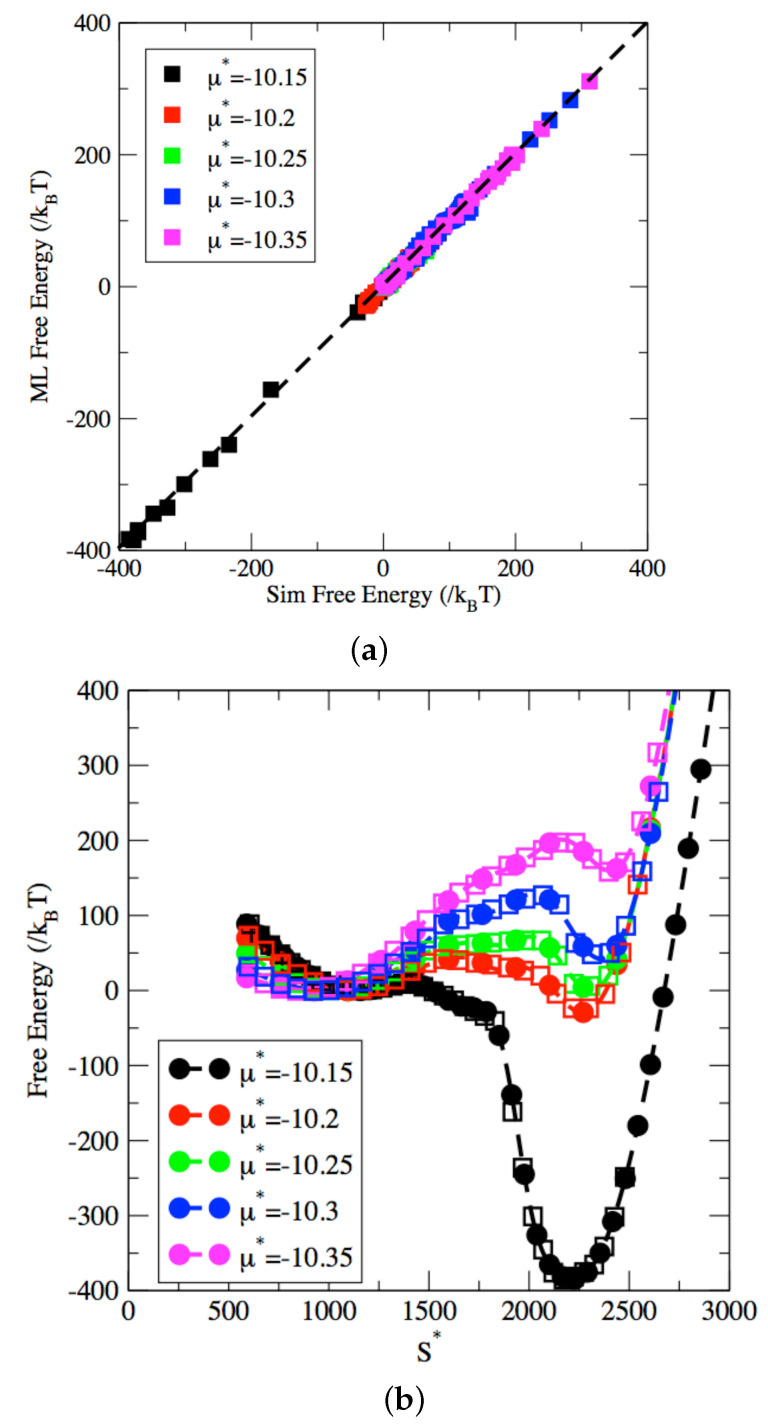
Comparison between free energy obtained with Machine Learning (ML) model (dashed line with circles) and from simulation results (squares) in a nanopore with a 12σ diameter. Results are shown for chemical potentials μ=−10.15 (black), μ=−10.2 (red), μ=−10.25 (green), μ=−10.3 (blue) and μ=−10.35 (pink). (Inset) performance of ML model against simulation results.

**Figure 7:**
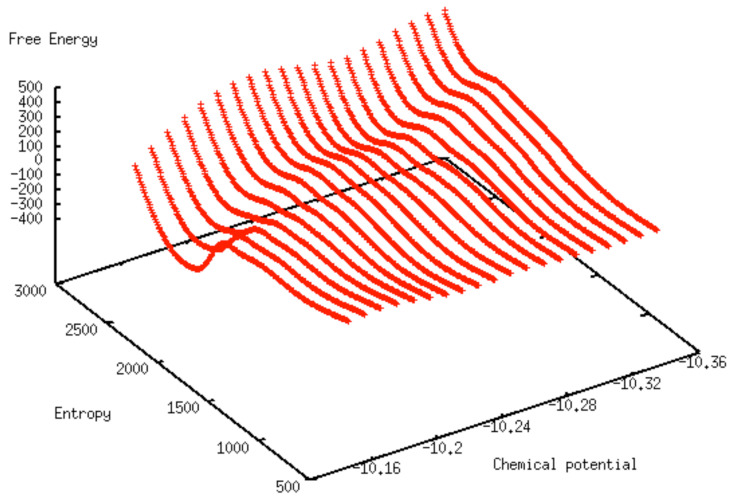
Free energy surface predicted by ML model for a pore width of 12σ.

We now turn to the prediction by ML models of the free energy profiles for a pore with a width of 12σ. In the next two figures, we present two different types of results. First, we show in Figure 6a comparison between simulation and ML results to validation the model plotted in Figure 6, i.e., that the ML model can model accurately the simulation results. Second, we use the ML model to extrapolate beyond the data and predict the entire free energy surface shown in Figure 7. As shown as the plot comparing the simulated free energy and the ML predicted free energy in Figure 6a, the ANN architecture provides an excellent model for the free energy of the adsorbed fluid during the capillary phase transition process. Furthermore, Figure 6b shows a direct comparison between the free energy profiles computed from the μVT-S simulations and the free energy profiles predicted by ML. This plot further established the reliability of the ML model to accurately account for the dependence of the free energy along the transition pathway, both for evaporation and condensation processes.

The ML predicted free energy surface is shown in Figure 7 over the $\left(S^{*},\mu^{*}\right)$ parameter space. As in the 2D free energy plots, and as a result of the training process, the origin for the free energy is set to that of the low-density, vapor-like phase. As expected, the ML free energy surface exhibits two troughs, corresponding to the two regions for which a free energy minimum can be reached. The two valleys are observed for varying values of $\mu^{*}$ and either for $S^{*}$ around 1000 for the low density, vapor-like adsorbed fluid, or for $S^{*}$ around 2500 for the high density, liquid-like, adsorbed fluid. The free energy minimum attached to the liquid-like fluid is found to steadily increase from a very low, and negative, free energy at high $\mu^{*}$ values (e.g., at −10.15) to a very high, positive, free energy at low $\mu^{*}$ values (e.g., at −10.35). In addition to yielding the free energy value for any set of $\left(S^{*},\mu^{*}\right)$, the ML predicted free energy surface provides a way to estimate rapidly the conditions of coexistence of the two phases for the adsorbed fluid, leading to an estimated chemical potential at coexistence of $\mu^{*}=-10.24$, consistently with the simulation results shown in Figure 2.

**Figure 8:**
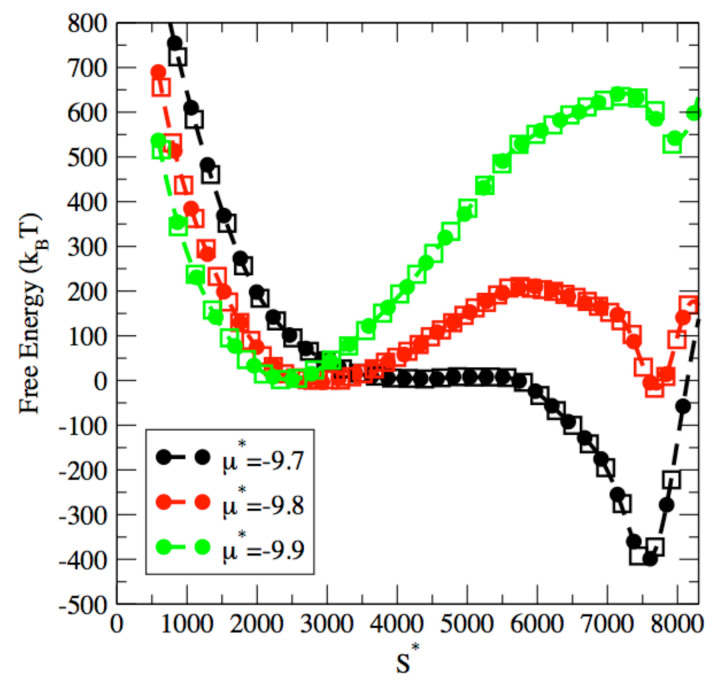
Capillary phase transitions in a pore with a width of 20σ: comparison between free energy obtained with ML model (dashed line with circles) and from the simulation results (squares). Results are shown for chemical potentials μ=−9.7 (black), μ=−9.8 (red) and μ=−9.9 (green).

We also test the transferability of the approach to other pore widths and show that an ML model with the same architecture, i.e., with 8 neurons per hidden layer, can also be optimized for a larger pore width such as 20σ. Indeed, Figure 8 shows that the simulated free energy profiles can be accurately modeled by ML, as evidenced by the very good agreement obtained between the simulations and the ML model for both capillary evaporation and capillary condensation processes.

## 4. Conclusions

In this work, we use molecular simulations and Machine Learning to study the capillary phase transitions that occur in a series of model mesopores, akin to MCM-41 as a function of pore width. To this end, we employ a recently developed molecular simulation technique that leverages entropy as a reaction coordinate for the transition process to shed light on the phase transition process. This allows to obtain the free energy profile corresponding to either the capillary evaporation process, from a pore containing a metastable high-density adsorbed fluid to a stable low-density adsorbed phase, or the capillary condensation process, that spans the pathway connecting a metastable, supersaturated, vapor-like, adsorbed phase to a stable liquid-like adsorbed phase. The results allow us to characterize the role played by intermediate states, which involve the formation of capillary liquid bridges and bubbles, and to analyze the dependence of the free energy barrier, as well as of the chemical potential that controls the coexistence of the two types of adsorbed phases. Furthermore, we propose a method to build Machine Learning models by optimizing Artificial Neural Network for the prediction of the free energy surfaces underlying capillary phase transition process in mesopores.

## Data Availability

The data presented in this study are available on request from the corresponding author.

## Abbreviations

ANN	Artificial Neural Network
COF	Covalent Organic Framework
MC	Monte Carlo
MCM-41 pore	silica mesoporous molecular sieve
ML	Machine Learning
MOF	Metal-Organic Framework
μPT-S	enhanced sampling simulations in the grand-canonical (μVT)
	ensemble using entropy (S) as reaction coordinate
NPT-S	enhanced sampling simulations in the isothermal-isobaric (NPT) ensemble using
	entropy (S) as reaction coordinate
